# Never tested for HIV in Latin-American migrants and Spaniards: prevalence and perceived barriers

**DOI:** 10.7448/IAS.16.1.18560

**Published:** 2013-05-09

**Authors:** Juan Hoyos, Sonia Fernández-Balbuena, Luis de la Fuente, Luis Sordo, Mónica Ruiz, Gregorio Barrio, María José Belza

**Affiliations:** 1 National Epidemiology Centre, Carlos III Health Institute, Madrid, Spain; 2 CIBER of Epidemiology and Public Health (CIBERESP), Spain; 3 Faculty of Medicine, Department of Preventive Medicine and Public Health, Madrid, Spain; 4 National School of Health. Carlos III Health Institute, Madrid, Spain; 5 Network of Addictive Disorders (RTA), Spain

**Keywords:** HIV, transients and migrants, Europe, Latin America, testing, barriers

## Abstract

**Introduction:**

Increasing the uptake of HIV testing and decreasing the number of undiagnosed people is a priority for HIV prevention. Understanding the barriers that hinder people from testing is vital, particularly when working with especially vulnerable populations like migrants. Most data available on migrants are based on African migrants in the UK, while barriers to HIV testing in Latin-American migrants living in Europe remain unexplored. Still, they account for a quarter of new diagnosis in Spain and suffer higher rates of delayed diagnosis.

**Methods:**

Between May 2008 and March 2011, a mobile unit offered free rapid HIV tests in different Spanish cities. We compared the prevalence of no previous testing, adjusting for potential confounders by two multivariate logistic models, and described differences in perceived barriers to testing in Latin-American migrants living in Spain versus Spaniards. Participants included men who have sex with men (MSM), men who have sex exclusively with women (MSW), and women.

**Results:**

Of the 5920 individuals who got tested and answered a self-administered questionnaire, 36.5% were MSM (20.4% previously untested), 28.9% were MSW (49% previously untested) and 34.6% were women (53% previously untested). Almost one quarter were Latin-American, of whom 30% had never been tested versus 45% of untested Spaniards. After adjusting for potential confounders, Spaniards were more likely to report no previous testing than Latin-Americans among women and MSW all together (Odds Ratio (OR)=2.0; 95% Confidence Interval (CI): 1.7–2.4) and among MSM (OR=1.6; 95% CI: 1.2–2.0). Among the 2455 who had never undergone an HIV test before, main barriers to testing were low perceived risk (54% Spaniards vs. 47% Latin-American) and concerns arising from the loss of anonymity (19.5% vs. 16.9%). Fear of rejection or discrimination and fear of legal problems were a barrier for <2%.

**Conclusions:**

Latin-American migrants living in Spain were more likely to get tested than Spaniards. Regardless of nationality, low perceived risk was the main barrier to testing whereas fear of stigma or discrimination and fear of legal problems were merely incidental. However, new Spanish austerity policies regarding healthcare for migrants in response to the economic crisis may reverse this situation.

## Introduction

Increasing uptake of HIV testing and decreasing the number of undiagnosed people is a priority for HIV prevention in Europe [1,2]. Delayed diagnosis is associated with a greater risk of HIV-related morbidity and mortality and with a poorer response to treatment. It also leads to higher viral loads which combined with a higher frequency of unprotected sex in those who remain undiagnosed [3], results in higher rates of HIV transmission [4–6]. Thus, delayed diagnoses translate into current and future higher costs to the health care system.

Of those at risk, migrants are particularly vulnerable to infection due to numerous social, economic, cultural, and legal factors [7–11] and the problem is compounded by their higher risk for late diagnosis [11–14]. In 2010, 49% of all new infections in Europe and 38% of all new infections in Spain were reported among migrants [15,16]. Within Europe, Spain is unique regarding HIV infection and migrants, since the majority of them, both in the general population and among HIV-infected, are Latin-Americans [7]. Whereas in Spain, 21% of new HIV diagnoses were among Latin-Americans and only 8% were among sub-Saharan migrants, Europe as a whole reported that 19% of new HIV cases were among sub-Saharan migrants and only 6% among Latino (41% of them reported by Spain). Further, the proportion of Latin-Americans diagnosed with HIV in Spain is on an upward trend, whereas the sub-Saharan representation among the HIV-infected remains stable [16].

Research examining HIV testing uptake and related barriers among migrant population in Europe is scarce [17]. According to the existing literature, the main barriers to testing among migrants and ethnic minorities include fear of HIV itself, of the legal and/or administrative impact of a positive test result, and of the related stigma and discrimination. Other important barriers involve a lack of cultural sensitivity, communication and language problems, low priority given to HIV, and lack of knowledge about health services [11]. However, most data available on migrants are based on African migrants in the UK [11], with little or no information about other migrant populations in other host countries. Moreover, most of the studies focus on untested or HIV-negative MSM (men who have sex with men) in the UK and the Netherlands, with no information collected on women or heterosexual men, whose contribution to the epidemic should not be ignored (in 2009, over 50% of new female HIV diagnoses in Spain were reported among migrant women [14].)

Based on sociological analysis [18], it may be argued that due to shared cultural and linguistic heritage, Latin-Americans living in Spain do not experience nearly the same barriers and constraints as non-Latino migrants or as many other migrants experience in other host countries. Further, starting in 2000, Spain granted migrants full access to Spanish subsidized health care services under the same conditions as Spaniards, regardless of legal status [19]. Whereas full access to care substantially reduced important barriers to HIV testing for all migrants, given the already lower barriers experienced by Latino-Americans in Spain, it is possible that this particular migrant population and Spaniards may encounter similar barriers to testing. Thus, Latino-American experience of HIV testing may be much closer to the Spaniard experience than to the experience of other migrants in Spain or most migrants in other host countries. However, this hypothesis remains unexplored.

In this article, we aim to compare the prevalence of no previous HIV test between Latin-American migrants and Spaniards who agreed to a rapid HIV test in a mobile unit while adjusting for potential confounders. Our second aim is to describe differences in perceived barriers to HIV testing across these migrants and Spaniards as well as across subpopulations defined by gender and sexual behaviours, i.e., MSM, MSW (men who have sex exclusively with women), and women.

## Methods

Between May 2008 and March 2011, a mobile unit offered free, rapid HIV testing in different areas throughout Spain: Madrid City, two working-class suburbs of Madrid, three coastal cities in the southeast, and in the Canary Islands. The Madrid city locations included a well-known gay neighbourhood and a neighbourhood with a high immigrant presence. In the two working-class suburbs cities, the van was located near railway stations and, finally, in the other cities the van was parked in busy, centrally-located streets with high pedestrian traffic.

At a manned desk next to the van, individuals interested in taking the test were given information about the characteristics, advantages, and limitations of rapid HIV testing. Those who decided to proceed signed an informed consent form and entered the van where a trained nurse/doctor completed a brief pre-counselling session. Blood collected via finger prick was tested with the *Determine*^®^*HIV*-1/2 test. While waiting for the test results (20 minutes), the subjects completed an anonymous self-administered questionnaire that was code-linked to their HIV test results. Because participants could only complete the questionnaire while sitting or standing on the street, the maximum length of the questionnaire was limited. Some questions were not available from the start of the study while other questions were asked only for certain time periods, all while keeping the length of the questionnaire constant. The survey collected socio-demographic data, sexual behaviours, and HIV testing history. For participants who had never been tested before, we tried to identify the main reason for never testing before through a multiple-choice question with 11 closed possible answers and one open-ended. A basic data collection sheet in English and French was available to those with low Spanish proficiency. This sheet included questions regarding basic socio-demographic and behavioural variables and the health professionals based on participants’ responses filled it out.

### Data analysis

Out of the 8076 individuals who underwent HIV testing, our analysis comprises the 5920 participants who met the inclusion criteria: being born in Spain or Latin-America and having answered the three key variables of interest (HIV testing history, gender/sexual behaviour, and reason for never testing, if applicable). Migrants were defined as having been born outside of Spain.

Taking into consideration the taxonomic utility of the variable gender and the classical transmission categories, a descriptive analysis of the participants’ characteristics was performed stratifying results by three categories: men who have sex with men (MSM), men who have sex exclusively with women (MSW), and women.

For both Latin-Americans and Spaniards, the percentage of no previous HIV test was calculated for each gender/sexual behaviour category. Based on the similarities revealed in the preliminary results and to control for potential confounders, we built two different multivariate logistic models, one for MSM alone and another combining MSW and women.

To explore the main barriers to testing, we performed a subgroup analysis including only those who did not have a previous HIV test (*n*=2455). The main reason for never testing was tabulated separately for Latin-Americans and Spaniards by the three categories of gender/sexual behaviour. Again, since MSW and women reported similar reasons for never testing, results are presented in two groups: MSM versus MSW and women. Comparisons between these two groups were tested for statistical significance using the Chi-square (*χ* ^2^) test.

To assess participant's behavioural risk level, we created an indicator variable classifying participants as: low-risk (never injected drugs and always used condoms with casual sexual partners, if any, in the last 12 months); or high-risk (ever injected drugs, have been paid for sex, or may not have always used condoms with casual partners in the last 12 months). Those who could not be classified due to missing data were grouped into a third, undefined, category. The institutional review board of the Health Institute Carlos III approved the study.

## Results

### Socio-demographic characteristics and risk behaviours

Of the 5920 respondents, 36.5% were MSM, 28.9% were MSW and 34.6% were women (Table 1). Spaniards and Latin-Americans accounted for 76 and 24%, respectively. Over half (53.7%) were under 30 years of age, with women being younger than men (63% of the women were under 30 versus 50% of the MSM and 48% of the MSW). A majority of the male participants classified as MSM based on their reported sexual relationships with men, self-identified as homosexual or bisexual. Approximately 9% stated that their sexual identity was heterosexual.

**Table 1:** Characteristics of Latin American migrants living in Spain and Spaniards who took a rapid HIV test in a mobile unit in Spain, 2008–2011

	MSM^a^ (*N*=2159)		MSW^b^ (*N*=1714)		Women (*N*=2047)		Total (*N*=5920)	
				
	*N*	%	*N*	%	*N*	%	*N*	%
Country of birth			***Sociodemographics***					
Latin-America	522	24.2	398	23.2	502	24.5	1422	24.0
Spain	1637	75.8	1316	76.8	1545	75.5	4498	76.0
Age								
<25	553	26.0	372	22.5	665	33.7	1590	27.6
25–29	504	23.7	422	25.5	576	29.2	1502	26.1
30 or older	1067	50.2	863	52.1	734	37.2	2664	46.3
With university degree	1155	53.9	704	41.3	1051	51.6	2910	49.5
Work as main source of income	1785	84.4	1460	87.2	1598	79.5	4843	83.5
Self-reported sexual identity								
Homosexual	926	79.4						
Bisexual	130	11.1						
Heterosexual	110	9.4						
Not in questionnaire^c^	951							
Relationship with gay culture								
Member of a gay CBO^d^	148	11.1						
Frequents gay scene, not member of a gay CBO^d^	867	64.7						
Not related to gay scene	324	24.2						
Not in questionnaire^c^	620							
			***Behavioral characteristics***					
Ever injected drugs	45	2.1	63	3.9	38	1.9	146	2.5
Unprotected sex with occasional partners last 12 months^e^	499	23.1	624	36.4	841	41.1	1964	33.2
Number of heterosexual partners last 12 months								
0–1			439	27.1	739	38.4		
2–4			765	47.3	839	43.6		
>5			413	25.5	345	17.9		
No of homosexual partners last 12 months								
0–1	403	19.4						
2–4	570	27.5						
5–9	424	20.5						
>10	676	32.6						
Internet as main source of meeting partners last 12 months	312	25.8						
Not in questionnaire^c^	951							
Self-reported STI								
Never diagnosed with a STI	787	45.4	940	73.6	1096	65.4	2823	60.2
Diagnosed within the last 12 months	216	12.4	55	4.3	129	7.7	400	8.5
Not in questionnaire^c^	424		436		370		1230	

a MSM: Men who have sex with other men.

b MSW: Men who only have sex with women.

c For some time, this question was not available in all questionnaries. Percentages calculated on respondents.

d CBO: Community Based Organization.

e Anal intercourse when referring to MSM; anal/vaginal intercourse when referring to women and MSW.

Unprotected sex with casual partners in the last 12 months was reported by 23% of MSM, 36% of MSW and 41% of women. In that same period, 53% of MSM, 26% of MSW, and 18% of women reported five or more sexual partners. Almost 40% of our participants have never been diagnosed with an STI; this proportion varies widely among MSM (55%), MSW (27%), and women (35%). Only 2.5% of the sample reported having ever injected drugs. Finally, we found no statistical differences in risk behaviours between Spaniards and Latin-American migrants (data not shown).

### HIV testing

**Figure 1:**
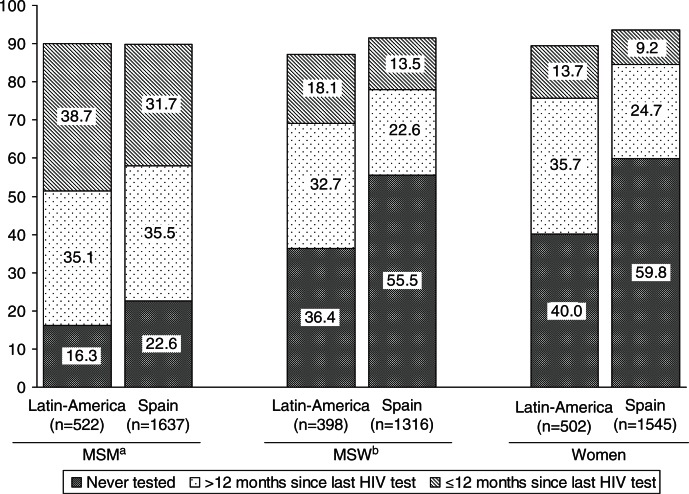
History of HIV testing in Latin Americans vs Spaniards by gender/sexual behaviour in people who undertook a rapid HIV test in a mobile unit in Spain, 2008–2011 (*n*=5920).* *Between 6% and 11% of the respondents had a previous HIV test but could not be specified when the last test was done. ^a^Men who have sex with men; ^b^Men who only have sex with women.

Over 40% of the participants (2455) had never undergone an HIV test before (45% of the Spaniards and 30% of Latin-Americans; p<0.0001) (data not shown). Women were the most likely to have never been tested for HIV (53%), followed by MSW (49%) and MSM (20.5%). In all three groups, the proportion of Spaniards reporting no previous HIV test was about 40–50% higher than for Latin-Americans (Figure 1).

Multivariate analysis showed that the likelihood of never being tested before was twice as high for Spaniards as for Latin-American migrants among women and MSW (_a_OR=2.0, 95% CI: 1.7–2.4). Among MSM, Spaniards were 60% more likely to never having being tested than Latin-American migrants (_a_OR=1.6, 95% CI: 1.2–2.0) (Table 2).

**Table 2:** Crude and adjusted association with not having undergone and HIV test before. Latin-Americans and Spaniards who took a rapid HIV test in a mobile unit. Spain, 2008–2011

	Men who have sex with men					Women & heterosexual men				
		
Country of birth	%	_c_OR	(95%CI)	_a_OR	(95%CI)	%	_c_OR	(95%CI)	_a_OR	(95%CI)
Latin America	16.3	1		1		38.4	1		1	
Spain	22.6	1.5	(1.2–1.9)	1.6	(1.2–2.0)	57.8	2.2	(1.9–2.6)	2.0	(1.7–2.4)

%, percentage of never testing; _c_OR, crude odds ratio; _a_OR, adjusted odds ratio; CI, confidence interval. Adjusted for: age, level of education, history of injecting drugs, number of partners in the last 12 months, having unprotected sex with occasional partners in the last 12 months, history of STI; in the model for men who had sex with men also for self-reported sexual orientation, relationship with gay culture and using Internet as the main source of meeting partners.

### Barriers to HIV testing

The top reason (well above the next main reason) provided by participants for never getting tested across geographic origin, gender, and sexual behaviour, was perceiving that they were at low risk for HIV. This perception derived from believing that their behaviours were not putting them at risk of infection (31.6%) or from feeling very healthy (21.3%) (Table 3). Spaniards were more likely to perceive themselves as being at low risk for HIV (54.3%) than Latin-Americans (46.7%) for MSM as well as MSW and women. Concerns arising from the loss of anonymity was the second most reported reason for not testing (19%) which, although similar across geographic origins, was slightly more relevant for Spaniards than Latin-Americans (19.5 vs. 16.9%, respectively).

**Table 3:** Self-declared reasons for never had undergone an HIV test before. Latin-American migrants vs. Spaniards who underwent a rapid HIV test in a mobile unit in Spain, 2008–2011

	MSM^a^ (*N*=455)		Women and MSW^b^ (*N*=2000)		Total (2455)	
			
	Latin-America (*N*=85)	Spain (*N*=370)	Latin-America (*N*=346)	Spain (*N*=1654)	Latin-America (*N*=431)	Spain (*N*=2024)
	%	%	%	%	%	%
I thought that with my behaviors I could not be infected	22.4	32.4	26.6	33.0	25.8	32.9
I found myself very healthy	18.8	15.7	21.4	22.7	20.9	21.4
Fear of the consequences for my health	20.0	11.1	12.1	10.4	13.7	10.5
I knew I had to wait several days to get the results	4.7	6.2	3.8	4.6	3.9	4.9
I wanted to do it in a private center and had no money	4.7	0.8	5.2	1.1	5.1	1.0
Concerns arising from the loss of anonymity^c^	14.1	19.2	17.6	19.6	16.9	19.5
Fear of rejection or discrimination	2.4	4.1	1.4	0.8	1.6	1.4
Fear of not being able to get /losing work or residence permit^d^	1.2	0.5	2.3	0.2	2.1	0.2
Others^e^	11.8	10.0	9.5	7.6	10.0	8.0

a Men who have sex with other men.

b Men who only have sex with women.

c Includes: “I didn't want to go to my GP/health center”, “did not know where to go without having to identify myself” and “discomfort when answering intimate questions”.

d Includes: “Fear of losing/preventing from finding a job”, “didn't had a residence/work permit and thought I would have problems to get it”.

e Replies impossible to assimilate in any of the other categories.

As barriers to HIV testing, fear of rejection or discrimination and fear of legal problems are almost anecdotal reasons (<2%). Latin-Americans reported being slightly more concerned than Spaniards about the consequences for health that a positive result could have (13.7 vs. 10.5%, respectively), but the gap doubles among MSM (20% of Latin-American vs. 11% of Spaniards). Further, Latin-Americans are more concerned about not having money for getting tested in a private institution than Spaniards (5.1 vs. 1.0%, respectively) (Table 3).

Based on self-reported risk behaviours, we classified 50% of the sample as high-risk for HIV and only 40% as low-risk individuals. The remaining 10% was unclassifiable due to missing data. Among high-risk participants, 46.5% reported perceiving they were at low-risk for infection (43.3% of Latin-Americans vs. 47.3% of Spaniards). When examined by gender/sexual behaviour, we observed that over half of high-risk MSW had a perception of being at low risk (51.6%) followed by women (44.4%) and MSM (41.6%).

## Discussion

To our knowledge, this study is the first of its kind to address differences in HIV testing and perceived barriers to testing between Latin-American migrants in Europe and native-born Europeans. Moreover, it examines a diversified population including not only MSM but also women and MSW. Inasmuch as the social, economic and political scene is changing in Spain and Europe, this work is necessary as a baseline to track changes in the social context and access to health services by Latin-American migrants.

This paper shows that Latin-American migrants residing in Spain are more likely to have ever been tested for HIV than the native-born. It also points out that the main reason that prevents Latin-Americans, regardless of gender and sexual behaviour, from undergoing an HIV test is the same as for Spanish: not feeling at risk for HIV. Finally, the study reveals that fear of rejection and discrimination and fear of legal or administrative problems due to getting tested is merely incidental in both Latin-Americans and Spaniards.

Past research describing the uptake of HIV testing among migrants [11], has also concluded that they were more likely to be tested than the general population. However, other studies have focused mainly on black Africans living in the UK [20,21], originally from areas with a high prevalence of HIV infection, and constituting the main targets of all testing programs. Thus, the higher prevalence of HIV testing among these migrant subpopulations compared to the host country population is to be expected. In contrast, this is not the case here since the group of migrants examined in our study do not come from countries with a high HIV prevalence [22].

Our findings support those of the only other study of Latin-American migrants in Europe, of which we are aware. This Portuguese study [8] determined that the rate of ever-HIV-tested among Latin-Americans was higher than the rate found by previous studies on the general Portuguese population. However, the authors were unable to compare their results against a similar Portuguese-born population or to account for the prevalence of risk behaviours. Because people demanding HIV tests usually have a higher prevalence of risk factors than average, comparisons with the general population make little sense conceptually speaking. For example, MSM are grossly overrepresented in our sample when compared to the general population (36 vs. 4%) [23] and not surprisingly, the prevalence of never testing for HIV among MSM is much lower than that of the general population [24–28]. In addition, the large size of the sample and the data collected on socio-demographic and behavioural characteristics allowed analyses to be adjusted for potential confounders.

However, our results should be interpreted in the context of the study's limitations since they are not drawn from representative samples of either the migrant or the general population of Spain. Because of this, we can not rule out the hypothesis of the existence of subgroups of HIV-infected Latin-Americans living in Spain, who have never been tested or have tested at a lower rate than Spaniards with the same behavioural characteristics. Nevertheless, it is worth noting that the only probability sample survey of the general population addressing this question in Spain also found a positive association between being a foreigner and having been tested for HIV [29]. Unfortunately, the probability survey's sample size of the foreign community was too small to warrant analyses of differences by geographical area of origin or to adjust for socio-demographic and behavioural factors, as we have done in the present study.

Understanding the barriers that hinder people from testing for HIV is vital to increase test rates. This is particularly relevant when working with especially vulnerable populations, like MSM and migrants. In our study, we found no differences between Latinos and Spaniards regarding the main barrier: low perceived risk for infection. The perception of risk of exposure to the disease is highly influential on an individual's decision to accept or seek an HIV test. Those who do not perceive themselves to be at risk will not ask for a test or even decline when offered one, resulting in many lost chances for early diagnosis [25,30–32].

It is worth mentioning that almost half (46.5%) of those who were classified as high risk for HIV based on their self-reported behaviours, still displayed a low-risk perception, regardless of geographic origin. Among MSW, the group with the highest rate of late diagnoses [16], this proportion was even higher (51.6%). This indicates deficient knowledge about the mechanisms of HIV transmission and, consequently, also about the use of effective protective measures [33].

We want to highlight that, even for Latin-American MSM (3.6%), the fear of rejection or discrimination and fears about legal or administrative status were barely mentioned as barriers for HIV testing. Other authors found rejection, social isolation, loss of social status and community support, etc. as the main reasons for not testing especially among migrants and MSM [2,11,31,34,35]. However, again, the main body of this literature examines black African migrants living in the UK [17,36,37], who may have similar concerns regarding many aspects of their daily lives and not only HIV testing. Perhaps the reason for these different types of fear is the greater cultural proximity between Spaniards and Latin-Americans than among migrants and locals in other host countries. Further, it could also derive from the discrimination and stigma attached to being MSM in many Latin-American countries, so that upon arrival to Spain, a country more accepting of homosexual behaviours, migrant MSM feel more integrated than in their country of origin. This may result in their degree of worry levelling with that of the Latin-American MSW and women.

In contrast, Latin-American MSM were more concerned about the health implications of testing positive than any other groups. As this is not linked to fears about legal or administrative status, as just discussed, it could be related to not receiving care if HIV-positive. However, this is not likely given that Spain has provided free antiretroviral treatment to all HIV patients regardless of legal or administrative status [7] until recent changes in the legislation discussed below. However, as aforementioned, any interpretation of our findings on barriers should take into consideration the absence of a probability sample.

Due to the financial constrains caused by the economic crisis, it seems that, from September 2012, migrants in irregular legal situations will only receive emergency medical care, forfeiting free access to regular health services. Although it is uncertain what impact this new policy may have on HIV testing, it is reasonable to assume that the current situation may change creating new restrictions and hurdles [19,38]. Therefore, due in part to its timing, this study may serve as a baseline measure against which to compare future HIV testing rates and, thus, assess the impact of the new regulations for migrants regarding access to health services implemented in 2012. Because of these policy changes, we foresee that future studies may not be able to reproduce our main findings.

## Conclusions

We found a higher prevalence of HIV testing among Latin-American migrants residing in Spain than among Spaniards. Both groups shared the same top barrier to testing (low perceived risk of infection), and an absence of fear of rejection or discrimination and fear of legal problems as important barriers to testing regardless of legal status, or sexual behaviour.

Based on our results, policymakers should bear in mind that equal access to health services for both migrants and Spaniards has yielded very satisfactory results in regards to uptake of HIV testing. The new restrictions on healthcare access for migrants in irregular legal situations could easily turn out to be a step back in the HIV prevention achievements observed to date in this vulnerable population.
